# When the most popular outcome measure becomes a problem: a critical perspective on PROMs in orthopedics

**DOI:** 10.2340/17453674.2026.47062

**Published:** 2026-10-28

**Authors:** Christian Fugl HANSEN, Michael Rindom KROGSGAARD

**Affiliations:** 1 Department of Orthopaedic Surgery, Bispebjerg and Frederiksberg Copenhagen University Hospital, Copenhagen; 2 Institute of Sports Medicine Copenhagen ISMC, Department of Orthopaedic Surgery, Bispebjerg and Frederiksberg Copenhagen University Hospital, Copenhagen; 3 Department of Clinical Medicine, Copenhagen University, Copenhagen, Denmark

Patient-reported outcome measures (PROMs) have transformed orthopedic research by giving patients a voice in evaluating treatment by transforming patient experiences into simple numbers. In that way, softer endpoints can be quantified and dealt with mathematically. PROMs are generally accepted as the most important outcome in clinical research. They serve as primary outcomes in randomized controlled trials (RCTs) and registries, and PROM scores influence guidelines, resource allocation, and treatment choices. However, it is a common misunderstanding to interpret a PROM score as a precise measurement simply because it is numerical. As PROMs are measurement instruments, their measurement properties need proper evaluation—a discipline in which most orthopedic PROMs struggle [1]. This profoundly affects what these PROMs can be used for.

The Oxford Knee Score (OKS) and Oxford Hip Score (OHS) illustrate the problem. Both are widely used 12-item arthroplasty PROMs, each reported as 1 total score. However, they contain 2 conceptually distinct constructs addressing pain and physical function, respectively. A sum score is only meaningful if all items measure the same construct. Pain and physical function are aspects of life that we as surgeons seek to improve, but, although connected, they are separate concepts. Treating them as one might be analogous to adding meters to kilograms—they are simply different units. Thus, once the content of a PROM has been established, evaluating the structure is the first and most important step in ensuring validity [2]. Nonetheless, the classical test theory (CTT) statistics commonly used for PROM validation (e.g., Cronbach’s alpha, convergent/concurrent validity, test–retest reliability) assume structural adequacy without testing it. Hence, even after a thorough validation using CTT, the PROM output might be reliable but not valid, and what the convenient numerical values reflect remains unclear.

In recent evaluations of the Danish OKS and OHS questionnaires, 2 of the best existing validity methods (confirmatory factor analysis [CFA] and Rasch analysis) failed to support that any of them can meaningfully be reported as 1 total score [3,4]. Separating pain and function improved the structure, but it was still not adequate. In addition, 24–28% of patients reached the maximum OHS score 1 year after surgery. This marked ceiling effect means that OHS has a very limited discrimination ability among patients with good outcomes—the interval in which different prostheses or techniques are often compared.

An example of why item response theory (IRT) models, such as the Rasch model, is important relates to PROM response categories. PROM responses are ordinal, meaning that differences between scores are not necessarily equal [5]. CTT methods nevertheless assume equal intervals: a 10-point improvement from 20 to 30 is treated as equivalent to an improvement from 60 to 70, but the two may represent different improvements for the patient. Rasch analysis, in contrast, tests this assumption. If data fits a Rasch model, responses can be located on an interval-level scale, with equal measurements across the scale. Orthopedic PROMs are rarely subjected to this analysis, and among those that have been, only a few have shown acceptable fit. Without evidence of interval-level measurement, analyses may look convincing, but neither Cronbach’s alpha nor test–retest reliability can rescue a scale with a flawed structure.

This is not just a psychometric problem. It heavily influences our clinical choices. Poorly targeted, multidimensional, or otherwise inadequate PROMs can dilute treatment effects and increase the risk of type II error in otherwise well-planned RCTs. In our analysis of 54 musculoskeletal RCTs, only 44% used the best available PROM as the primary outcome. Trials using these “most appropriate” PROMs reported between-treatment differences in 46% of cases, compared with 22% when a less adequate PROM was used [6]. This finding cannot prove causality, but it is consistent with measurement theory.

O’Hara et al. recently demonstrated how outcomes can be diluted and hide important differences even when a PROM is responsive [7]. In a simulated tibial fracture trial of 400 patients, a reduction in nonunion from 20% to 10% using a new treatment was detected in approximately 81% of trials using nonunion as the endpoint, but it was only superior in 4.2% when the Patient Reported Outcomes Measurement Information System – Physical Function was used as the outcome. A new treatment may therefore be important for a group of patients while barely shifting the group mean PROM score, in particular if the PROM has poor validity, poor targeting, or ceiling effects. Consequently, the best treatment is not implemented.

The minimal clinically important difference (MCID) is another estimate that can severely distort the interpretation of study results. A statistically significant difference is often dismissed as clinically irrelevant when it does not cross a certain MCID. However, MCID is not a quality of the PROM and is heavily population-, context-, and anchor-dependent [8], with no fixed MCID for a given PROM. Solomito et al. calculated MCIDs for KOOS JR and HOOS JR using 16 accepted methods and obtained ranges of 0.5–36.6 and 0.6–34.3 points, respectively [9]. Which figure do we trust? Furthermore, applying MCID to PROM scores assumes that the score measures equally across the scale. Unless the scale has demonstrated interval-level measurement through Rasch analysis, that assumption is not supported [10].

This is problematic, particularly when an OHS MCID of, for instance, 5 points is used for evaluating the effect of total hip replacements. If one-quarter of patients already occupy the maximum score and many others cluster immediately below it, a 5-point improvement is impossible to reach for a substantial proportion of the cohort. Failure to exceed MCID may then reflect the quality of the instrument rather than the absence of meaningful treatment benefits. Currently, there is no consensus on a better way to calculate and interpret a clinically meaningful change. If conducting a prospective cohort study or RCT, we suggest adding anchor questions at different time points, making it possible to calculate MCIDs within your specific trial cohort.

The solution to these major problems is not to abandon PROMs. Patient experience is indispensable. Rather, we should stop granting PROMs methodological immunity. We would not accept a laboratory test with uncertain calibration simply because it is widely used. Patients deserve the same scrutiny to be applied to PROMs before they are approved for general use.

Before a PROM determines the primary conclusion of a trial, its content should be relevant to the target patients, its dimensional structure should be supported by modern test theory (CFA/IRT/Rasch), and its targeting and responsiveness should match the clinical question [2]. Where no adequate PROM exists, the limitation should be clearly acknowledged, and complementary clinical endpoints considered (e.g., anchor questions). Journals, reviewers, registries, and guideline panels should ask not merely whether a questionnaire has been labelled “valid and reliable,” but whether there is adequate evidence that the score used in this population functions as a measurement. Most “valid and reliable” statements are incorrect.

We increasingly use PROMs to decide which treatments work, which innovations merit funding, and how healthcare resources should be allocated. That makes measurement quality more, and not less, important. Patient-reported outcomes should influence orthopedic care. But a patient-reported number is not automatically a valid measurement, and precision in calculation cannot compensate for imprecision in the ruler.
